# Supplementary material for: Divergent confidence intervals among pre-specified analyses in the HiSTORIC stepped wedge trial: An exploratory post-hoc investigation
Source: PLoS One. 2022 Jul 1;17(7):e0271027. doi: 10.1371/journal.pone.0271027 (PMC9249209; doi:10.1371/journal.pone.0271027)
Supplement: S1 File — (PDF) [file pone.0271027.s001.pdf]

Simulation analysis

The divergent results immediately called into question the primary analysis model for the binary outcome. Preparatory simulation work had already been done when planning the study to determine sample size, but only statistical power had been considered. Also, several design parameters were uncertain at the time of the original calculations (e.g. outcome proportion, and strength of the time trend or seasonal effect).

We conducted further simulation analysis within R software [1] using parametric models based on the final study design and what we observed in the actual trial. Only one design scenario was considered: based on the actual HiSTORIC study design and primary analysis model results. Details of the simulation method are presented in S1 Table.

S1 Table: Simulation analysis method

**Data generating mechanism**	Simulation of the binary safety endpoint for 31,492 patients using a binomial distribution (the function “rbinom” in R) with probability of outcome dependent on the downward time trend observed in the HISTORIC study. At the midpoint of the study, the probability of outcome was set to be 0.036, and this varied from between 0.090 down to 0.014 depending on the time since the start of the study. The primary model results showed no significant seasonal effect for the binary safety endpoint, so the probability of outcome was not set to vary by season. Times of patient presentation were randomly sampled from a number sequence from 0 to 731 (representing days from the start of the study). Patients were assigned to one of seven sites with probabilities equal to the site (sample size) proportions to emulate the distribution of site sizes observed in the real trial. Sites were randomised to cross over to start the intervention at the beginning of one of 5 months within the randomisation period, again to mirror what happened in the real trial where sites did not adhere to the planned randomisation schedule.
**Analysis Models**	Two separate analysis models were fitted to the data: the primary analysis model and calendar matched analysis models, which were equivalent to those used in the real trial.
**Performance measures considered**	Type 1 error rate, 95% confidence interval width, bias, empirical SE, and model SE.
**Number of simulations**	Two thousand simulated trials were run to ensure that the standard error associated with the estimated Type 1 error rate was below 0.005. Formally, this called minimising the Monte Carlo standard error of the rejection percentage.[2]

Results of the simulation analysis are shown in S2 Table.

S2 Table: Summary of performance characteristics for each analysis model

	**Type 1 error rate**	**Mean bias (odds ratio)**	**Empirical Standard error**	**Mean model Standard error**	**Mean 90% CI width**
Primary analysis	0.044	1.02	0.14	0.14	0.46
Calendar matched analysis	0.055	1.00	0.09	0.09	0.28

The Type 1 error rate was reasonable for both analyses, and mean bias was low. However, precision of estimation was poor for the primary analysis as shown by the large standard errors and confidence interval width. In contrast, precision of estimation was much better for the calendar-matched analysis, but this analysis was of course more susceptible to confounding bias due to natural changes over time.

References

1. R Core Team (2019). R: A language and environment for statistical computing. R Foundation for Statistical Computing, Vienna, Austria. URL https://www.R-project.org/.

2. Morris TP, White IR, Crowther MJ. Using simulation studies to evaluate statistical methods. *Statistics in medicine*. 2019; 38(11):2074-102.
