# Supplementary material for: Divergent confidence intervals among pre-specified analyses in the HiSTORIC stepped wedge trial: An exploratory post-hoc investigation
Source: PLoS One. 2022 Jul 1;17(7):e0271027. doi: 10.1371/journal.pone.0271027 (PMC9249209; doi:10.1371/journal.pone.0271027)
Supplement: Attachment — Submitted filename: Response to reviewers.docx [file pone.0271027.s002.docx]

**We would like to thank the associate editor and reviewers for their constructive comments, which we are pleased to address in the revised version of the manuscript. We give a point-by-point response to the associate editor and reviewers’ comments below:**

Journal Requirements:

When submitting your revision, we need you to address these additional requirements.

1. Please ensure that your manuscript meets PLOS ONE's style requirements, including those for file naming.

**Thank you for your comment. We have now modified the manuscript so that it meets the PLOS ONE style requirements.**

2. Please provide additional details regarding participant consent. In the ethics statement in the Methods and online submission information, please ensure that you have specified (1) whether consent was informed and (2) what type you obtained (for instance, written or verbal, and if verbal, how it was documented and witnessed). If your study included minors, state whether you obtained consent from parents or guardians. If the need for consent was waived by the ethics committee, please include this information.

**Participant consent was not sought for our study and this was approved by the NHS Scotland Public Benefit And Privacy Panel For Health And Social Care (NHSS HSC-PBPP) committee. We have now clarified this in the methods section (page 5): “We received approval to use routinely collected National Health Service (NHS) data for research without the need for individual patient consent. This approval was granted by the NHS National Services Scotland Privacy Advisory Committee (PAC), now called the NHS Scotland Public Benefit And Privacy Panel For Health And Social Care (NHSS HSC-PBPP).”**

Reviewers' comments:

Reviewer #1: Several post-hoc statistical models were fitted for the primary outcomes of length of hospital stay and safety events. Models were adjusted for exposure time and incorporated spline fitting and a random effect for the time effect. The methods used were direct inclusion, regression adjustment for propensity score, and weighting, as well as a data augmentation approach. The new statistical models confirmed the results of the pre-specified trial analysis where the event rate was low: 0.36%.

Minor revision:
Table 1: In the “Primary analysis” specify the type of statistical model used for the analysis.

**Thank you for your comment. We have now specified that we used mixed effects models in the first row (below the headings) of Table 1. We have also clarified this in Table 2.**

Reviewer #2: Parker and colleagues should be congratulated for their efforts carrying out this important analysis. In their current paper, they investigate in very carefully robust analysis the reasons for the divergent results that were reported in the HiSTORIC trial. The current paper helps understand the results of the HiSTORIC trial more, and can assist researches with the design and implementation of future stepped-wedge cluster randomised trials. Parker and colleagues have confirmed the results of the pre-specified trial analysis and have validate their complexity.

**Thank you very much for these comments!**

Several major comments:
1. Although the design and the execution of the current analysis are very elegant, the additive scientific value is not well presented. The conclusion at the end of the manuscript is very long and should be shortened and more focused.

**Thank you for your comment. We have now made the conclusions section more focussed and have reduced its length by 117 words.**

2. Overall the discussion and the conclusion are very long and it's hard to follow. Would highly recommend to be more focused.

**Thank you for your comment. We agree, and have removed a substantial amount of material from the discussion section, including our long list of recommendations, which we felt was overly repetitive and not very focussed. As a whole, the length of these sections has reduced by almost 400 words, despite us adding in more material in response to your other helpful comments regarding including “explanations from the results section” and also including a strengths and limitations sections. We have made substantial changes to the discussion and conclusions sections so that they are now more focussed and easier to read.**

3. In the discussion the authors discuss several potential reasons for the differences between certain results under separate headings. I would recommend to choose two or three that the authors believe are the most important ones, expand on them, and mention the rest briefly. It's difficult for a non-statistician to understand which reason affected the diversity the most.

**Our changes (mentioned above in response to point 2) have helped to address these concerns and have made the discussion section more focussed. In particular, we have reduced the number of headings we are using in the discussion section. Our main conclusions regarding the reason for differences between results are highlighted in the Conclusions section (as well as covered in the section entitled “Horizontal versus vertical bias”). In the conclusions section we write: “we think a combination of issues jointly contributed to the divergent results: sparse event data, low number of sites, short randomisation phase, and non-adherence to the planned intervention schedule.”, and then go on to expand briefly on the key issues of sparse event data (in combination with low number of sites) and strong influence of data near the crossover points.**

Minor comments:
1. Limitation. Each study including the current one is prone to several limitations. I would highly recommend mention the limitation of the current study: post hoc; selective population etc..

**Thank you for your comment. We agree, and have now added a limitations section to the Discussion. In addition, we feel that the HISTORIC trial had important strengths that we need to acknowledge, and so we have also mentioned these in a “strengths” section.**

2. Figure labels for the ratio and the CI should be added.

**Thank you for your comment. We have now added the clarifying information (“geometric mean ratio” and “95% confidence intervals”) to the figure labels.**

3. The Results section contains many explanations for the findings. These should be moved to the discussion. The Result section should report findings only and not their interpretations or explanations.

**Thank you for your comment. We have moved explanations for the findings contained in the results section to the Discussion section (particularly the last two paragraphs of the results section).**
