# Supplementary material for: Divergent confidence intervals among pre-specified analyses in the HiSTORIC stepped wedge trial: An exploratory post-hoc investigation
Source: PLoS One. 2022 Jul 1;17(7):e0271027. doi: 10.1371/journal.pone.0271027 (PMC9249209; doi:10.1371/journal.pone.0271027)
Supplement: Attachment — Submitted filename: Responses to reviewer R2.docx [file pone.0271027.s003.docx]

**We would like to thank the reviewer for their comment, which we are pleased to address in the revised version of the manuscript.**

Reviewer #1: The results and conclusions from a relatively new study design are now more succinctly and clearly summarized.
Minor Revision:
State the underlying covariance structures used in the mixed effects models and the criteria for selecting them.

**Thank you for your comment. We have now specified in the paper that a “compound symmetry variance-covariance structure was assumed” for the linear mixed effects models (page 6).**

**We have also added a limitation to the limitations section of the discussion section (pages 17-18):**

**“Furthermore, the statistical models were fitted using the lme4 package [9] in R software [24], which makes the assumption of a simple correlation structure of compound symmetry for linear mixed effects models (i.e. no autocorrelation in model residuals). Although we attempted to fit models assuming autoregressive or general (unstructured) correlation using the nlme package [31], the models would not fit, presumably for the same reason as for the GEE model: the within-cluster sizes were too large. Nevertheless, we believe that compound symmetry is a reasonable assumption given that independent individuals attending emergency departments within each site may be expected to be approximately equally correlated (or uncorrelated) with each other conditional on the fixed effects including season and time of presentation. Indeed, a few of the models fitted include sophisticated modelling of the time trend (e.g. via splines), which we think makes the compound symmetry assumption even more plausible because residual autocorrelation due to incomplete modelling of the time trend is less likely.”**

**We confirm that a reference to the “nlme” package has now been added to our reference list (number [31]).**
